# Supplementary material for: Mapping the ultrafast flow of harvested solar energy in living photosynthetic cells
Source: Nat Commun. 2017 Oct 17;8:988. doi: 10.1038/s41467-017-01124-z (PMC5715167; doi:10.1038/s41467-017-01124-z)
Supplement: Supplementary file 1 — Supplementary Info [file 41467_2017_1124_MOESM1_ESM.pdf]

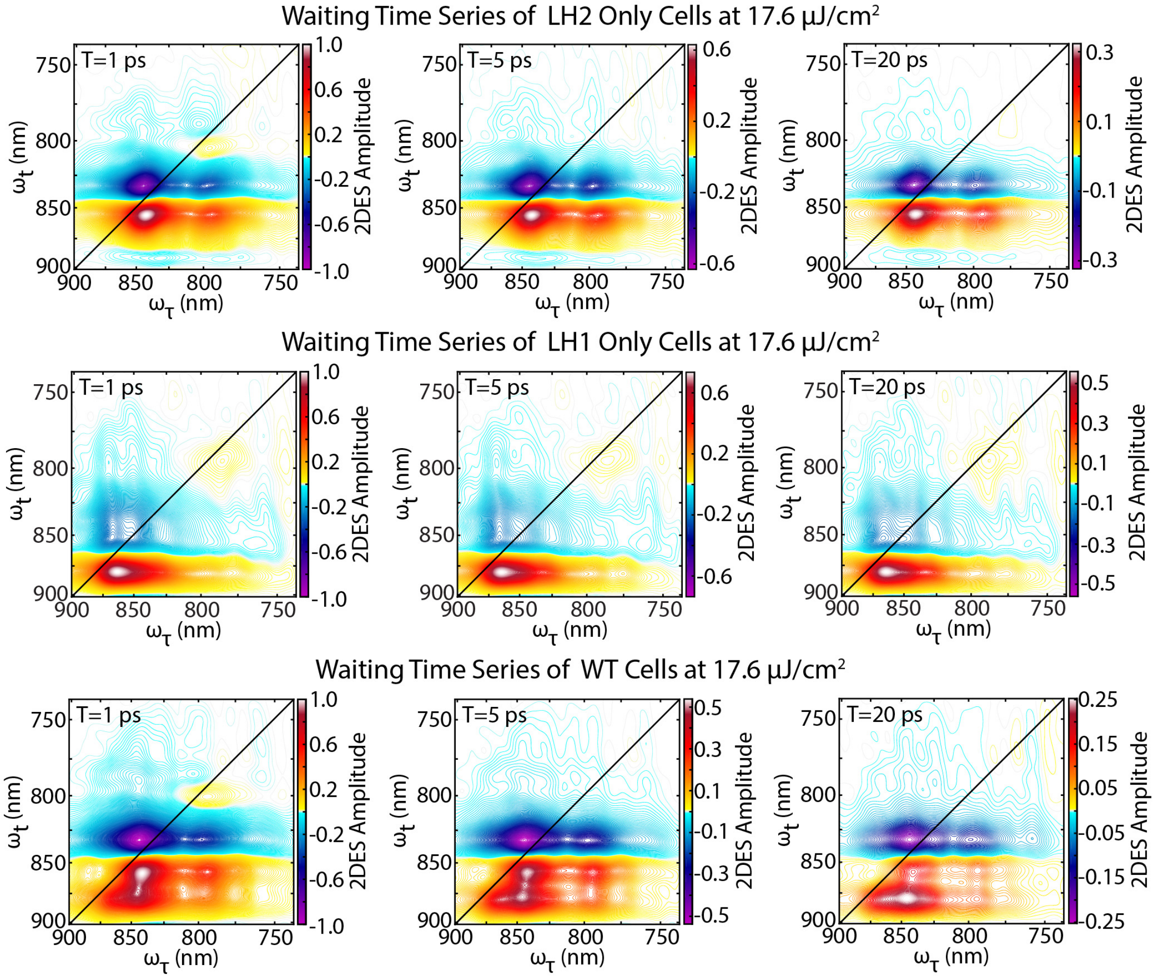

Supplementary Figure 1: Waiting time series of absorptive spectra from cells containing only LH2 (*top*), cells containing only LH1 (*middle*), and wild type cells (*bottom*) taken at 17.6 μJ cm^{-2}. The spectra show significant overlap between LH2 and LH1, as well as clear energy transfer cross peaks between LH2 and LH1 in the wild type cells at T = 5 ps and T = 20 ps.

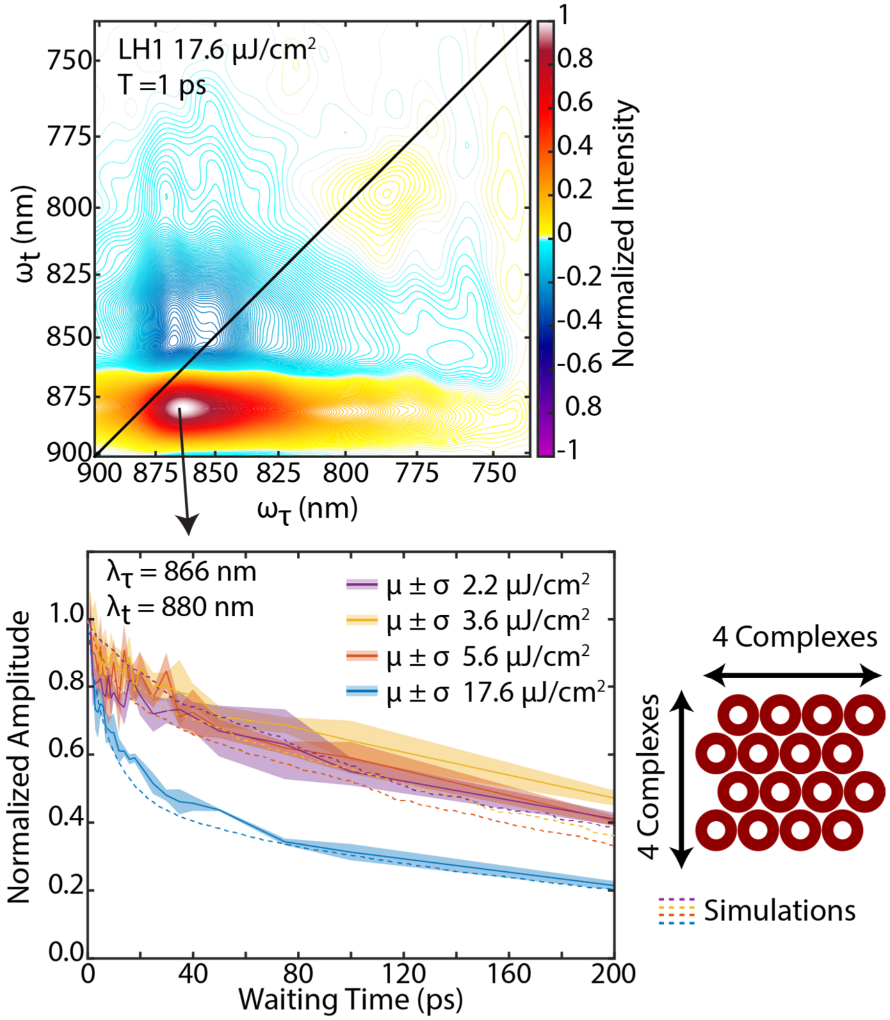

Supplementary Figure 2: *(top)* Absorptive 2DES spectrum of LH1-only cells taken with 17.6 μJ cm^{-2} at T = 1 ps. *(bottom)* Waiting time traces taken from the spectral location indicated acquired at different powers. The traces are the average of three scans and the shaded background represents ± the standard deviation. The change in dynamics with power is indicative of exciton-exciton annihilation. The dashed lines show agreement with a model membrane with LH1 domain sizes of 16 complexes, a transfer time of 4.7 ps, and an excited state lifetime of 250 ps.

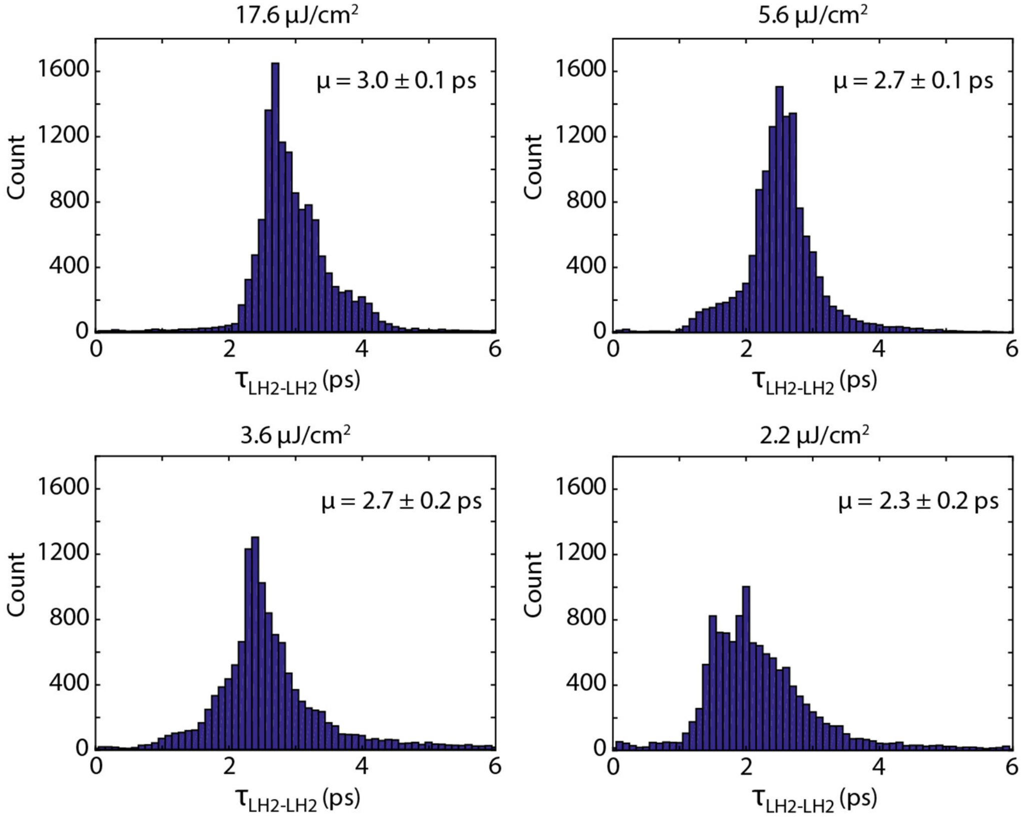

Supplementary Figure 3: Histogram of the energy transfer times between LH2s recovered from 2DES data of LH2-only cells at fluences of 17.6, 5.6, 3.6, and 2.6 μJ cm^{-2}.

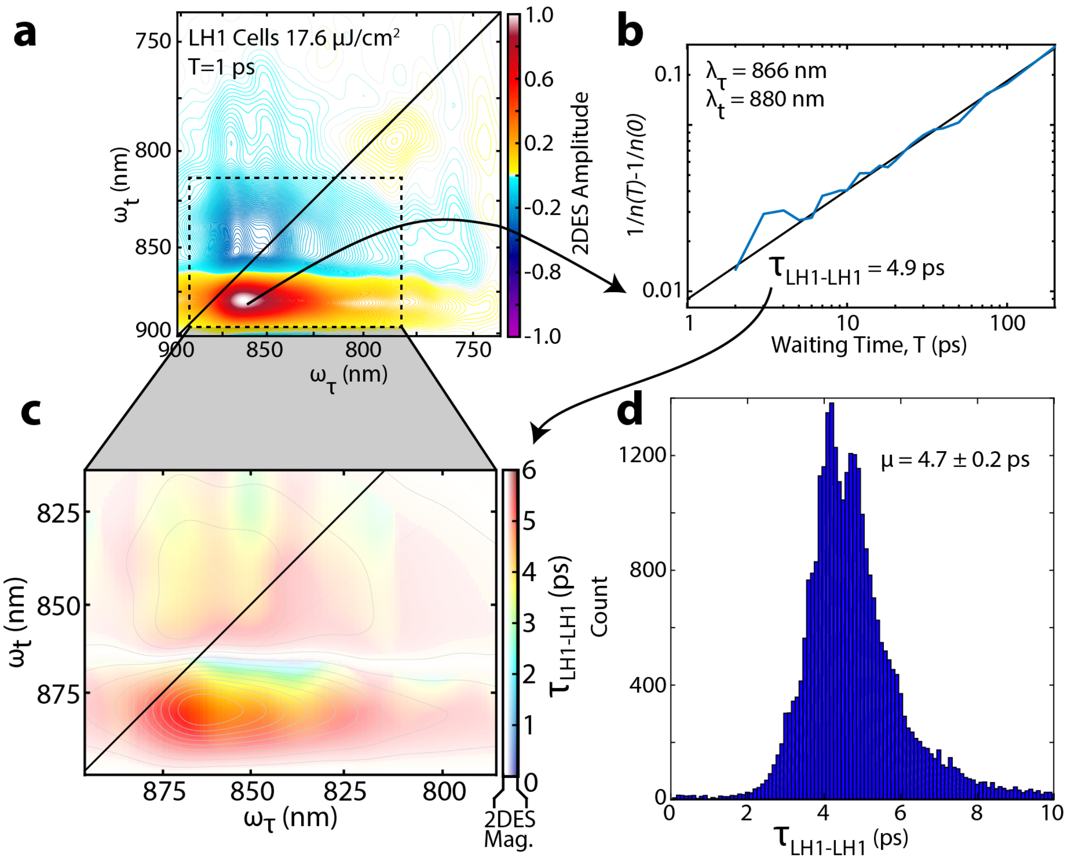

Supplementary Figure 4: **a** Absorptive 2DES spectrum of LH1-only cells at T = 1 ps collected at 17.6 μJ cm^{-2}. The dashed box is analyzed further for the lifetime of energy transfer between LH1 complexes. **b** Waiting time dynamics from the maximum ground state bleach and stimulated emission feature presented following equation 2, where the 2DES intensity is *n(T)*. The intercept of the linear relationship is used to retrieve the annihilation rate, γ_0, which in turn is used to recover the hopping time, τ_{hop}, given in equation 1. **c** Color map of the recovered τ_{hop}. The contours and saturation of the color is given by the intensity of the 2DES signal at 1 ps. **d** Histogram of the lifetimes recovered in **c** giving a mean of 4.7 ps for the lifetime of energy transfer between LH1s.

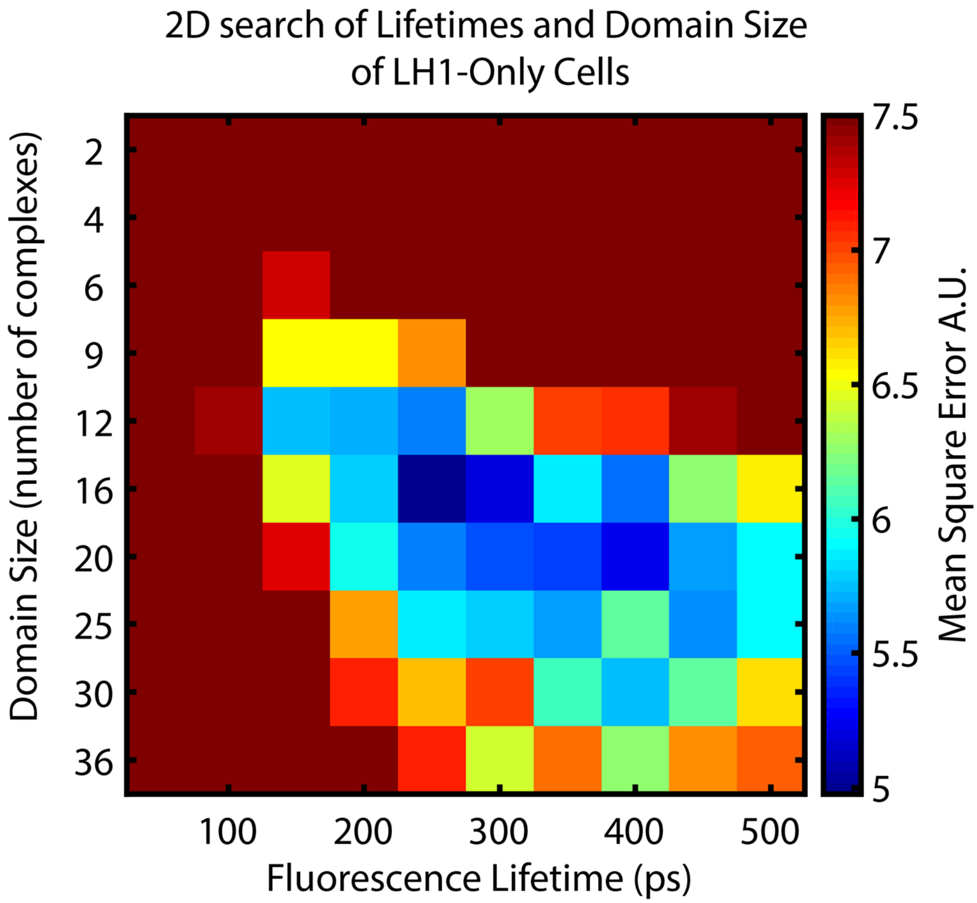

Supplementary Figure 5: Comparison of dynamics in LH1-only cells and LH1-only model membranes with varying domain size and fluorescence lifetime. The best fit is found with 16 complexes and a fluorescence lifetime of 250 ps. The comparison of the experimental data and a model with these optimal parameters can be seen in Figure S2.

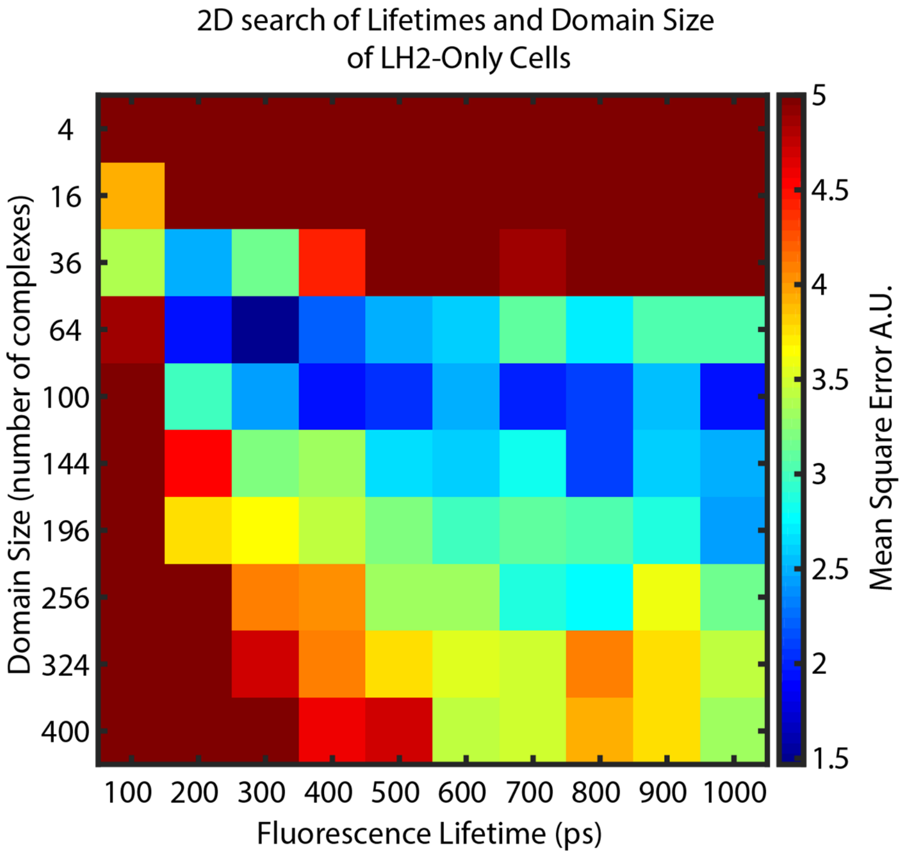

Supplementary Figure 6: Comparison of dynamics from LH2-only cells and LH2-only model membranes with varying domain size and fluorescence lifetime. The best fit is found with 64 complexes and a fluorescence lifetime of 300 ps. The comparison of the experimental data and a model with these optimal parameters can be seen in Figure 1**d**.

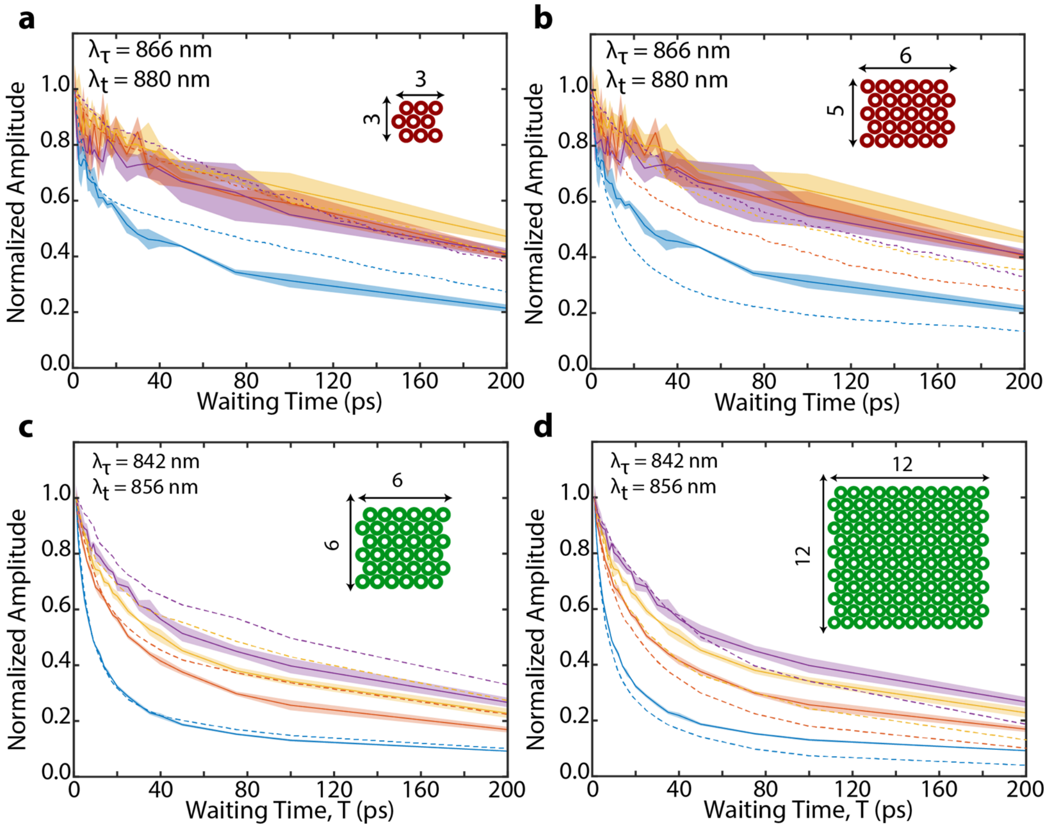

Supplementary Figure 7: Comparison of experimental dynamics to simulated dynamics recovered from model membranes with a 250 ps fluorescence lifetime and varying domain sizes in LH1-only (**a** and **b**) and LH2-only (**c** and **d**) cells. The clear deviation from the model is indicative of the tight constraint on domain sizes in both LH1- and LH2-only cells.

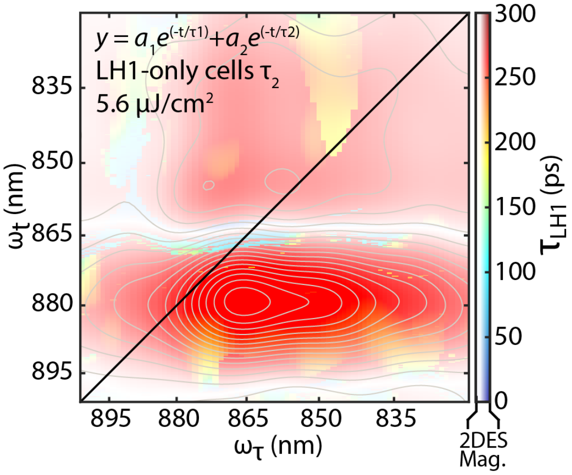

Supplementary Figure 8: Color map of the second lifetime from the biexponential fit. This lifetime in the LH1-only cells corresponds to the lifetime of the excited state in LH1 in the absence of the RC trap and annihilation. The saturation of the color as well as the gray contours are given by the intensity of the 2DES spectrum of LH1-only cells at T = 50 ps.

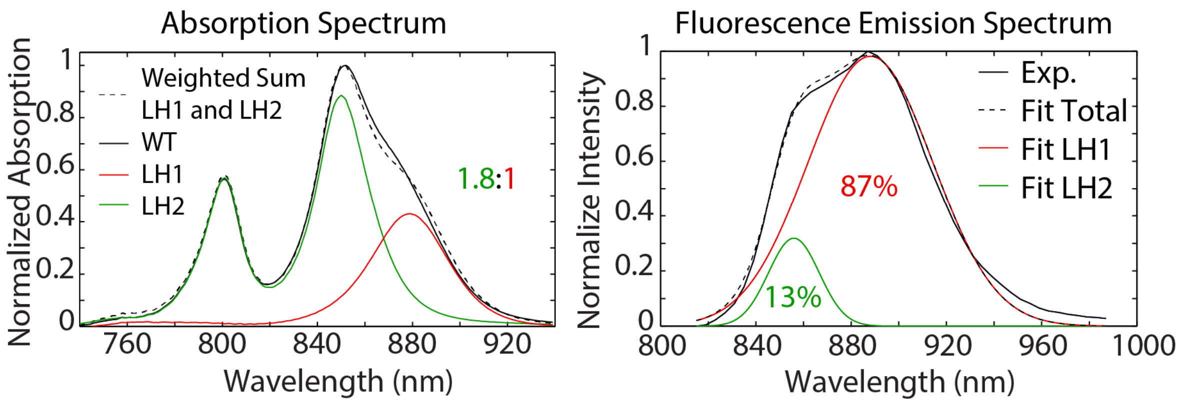

Supplementary Figure 9: (*left)* Absorption spectra of WT, LH1-only, and LH2-only cells with the scatter removed by fitting a quadratic function to long wavelengths. The dashed line is the result of a weighted sum fit to the WT absorption spectrum revealing a ratio of 1.8 LH2 to LH1. *(right)* Fluorescence emission spectrum from WT cells excited at 800 nm. The relative ratio between fluorescence intensity of LH2 and LH1 was determined by fitting two Gaussian functions to the spectrum and revealed that about 13% of the fluorescence was from LH2. This was used to constrain the back transfer rate from LH1 to LH2 to about 120 ps.

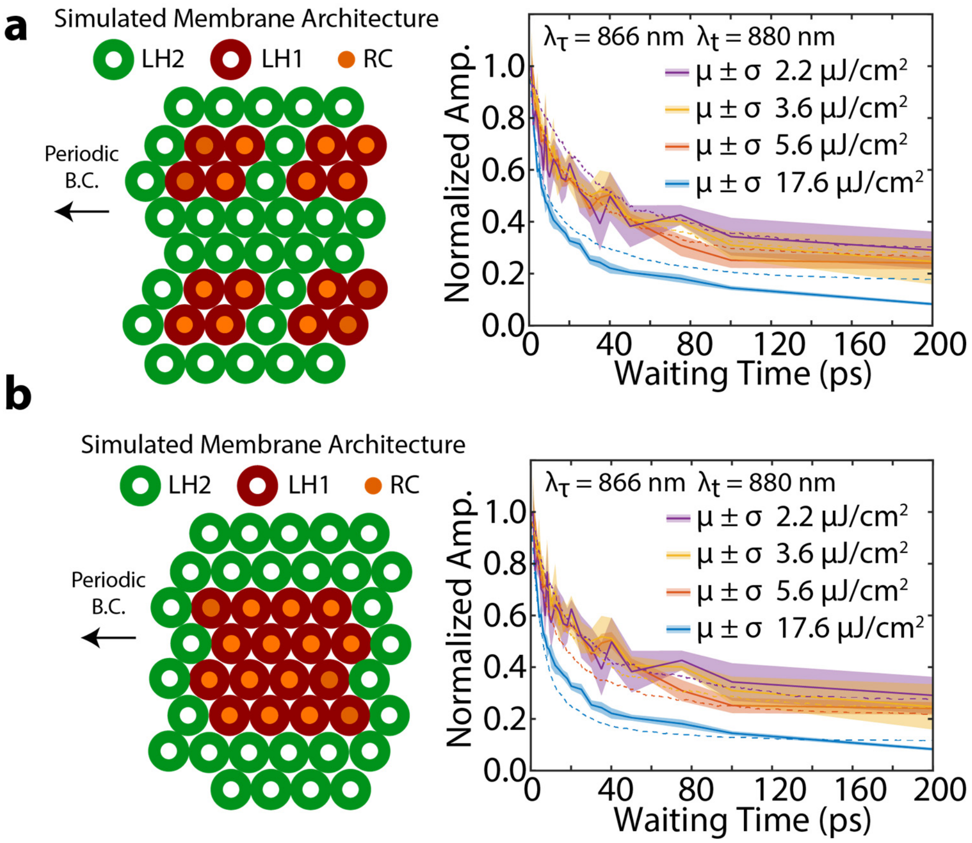

Supplementary Figure 10: Comparison of experimental data from the stimulated emission and ground state bleach peak of LH1 to dynamics recovered from model WT membranes with LH1 domain sizes of **a,** 4 and **b,** 16. The deviation from the model is indicative of the tight constraint on LH1 domain sizes in WT membranes.

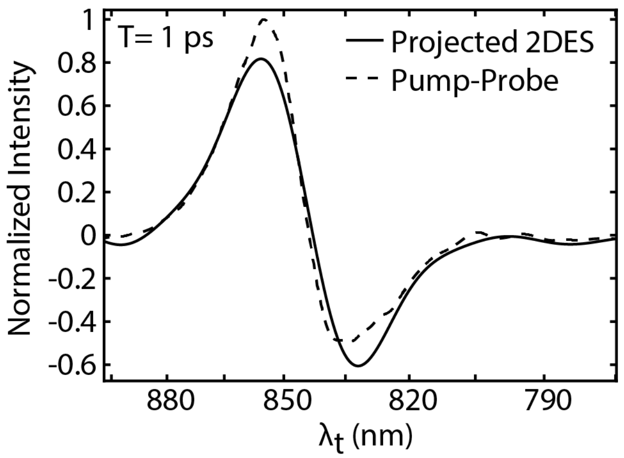

Supplementary Figure 11: Example of the phasing procedure showing the projection of the rephasing 2DES spectrum of LH2-only cells (*solid)* to the pump-probe spectrum (*dashed*) at T = 1 ps.

Complex-Complex	τ_{hop} (ps)
LH2→ LH2	2.7 ± 0.1
LH2→ LH1	4.8 ± 0.2
LH1→ LH1	4.7 ± 0.2
LH1→ RC	49 ± 3
LH2 fluorescence	273[1]
LH1 fluorescence	264 ± 7

Supplementary Table 1: Energy transfer times between complexes recovered from annihilation studies in mutant *Rba. sphaeroides* and biexponential fits to low fluence scans. Because annihilation was present at all powers in the LH2-only cells, the fluorescence lifetime of LH2 from Hunter et al.[1] was used for modeling.

Supplementary Reference:

1 Hunter, C. N., Bergstrom, H., van Grondelle, R. & Sundstrom, V. Energy-transfer dynamics in three light-harvesting mutants of Rhodobacter sphaeroides: a picosecond spectroscopy study. *Biochemistry* **29**, 3203-3207, doi:Doi 10.1021/Bi00465a008 (1990).
